# Longitudinal variations of brain functional connectivity: A case report study based on a mouse model of epilepsy

**DOI:** 10.12688/f1000research.6570.2

**Published:** 2015-07-16

**Authors:** A. Erramuzpe, J. M. Encinas, A. Sierra, M. Maletic-Savatic, A.L. Brewster, Anne E. Anderson, S. Stramaglia, Jesus M. Cortes

**Affiliations:** 1 Biocruces Health Research Institute, Cruces University Hospital, Barakaldo, 48903, Spain; 2 Achucarro Basque Center for Neuroscience, Zamudio, 48170, Spain; 3 University of the Basque Country (UPV/EHU), Leioa, 48940, Spain; 4 Ikerbasque: The Basque Foundation for Science, Bilbao, 48013, Spain; 5 Neurological Research Institute, Baylor College of Medicine, Houston, Texas, 77030, USA; 6 Dipartimento di Fisica, Universita degla Studi di Bari and INFN, Bari, 70125, Italy; 7 BCAM, Basque Center for Applied Mathematics, Bilbao, 48009, Spain

**Keywords:** Longitudinal study, brain functional connectivity, mouse model, temporal lobe epilepsy, mouse brain connectivity

## Abstract

Brain Functional Connectivity (FC) quantifies statistical dependencies between areas of the brain. FC has been widely used to address altered function of brain circuits in control conditions compared to different pathological states, including epilepsy, a major neurological disorder. However, FC also has the as yet unexplored potential to help us understand the pathological transformation of the brain circuitry. Our hypothesis is that FC can differentiate global brain interactions across a time-scale of days. To this end, we present a case report study based on a mouse model for epilepsy and analyze longitudinal intracranial electroencephalography data of epilepsy to calculate FC changes from the initial insult (status epilepticus) and over the latent period, when epileptogenic networks emerge, and at chronic epilepsy, when unprovoked seizures occur as spontaneous events. We found that the overall network FC at low frequency bands decreased immediately after status epilepticus was provoked, and increased monotonously later on during the latent period. Overall, our results demonstrate the capacity of FC to address longitudinal variations of brain connectivity across the establishment of pathological states.

## Introduction

Functional Connectivity (FC) quantifies the statistical similarities between brain areas ^1^. FC measures the influences between areas originated by different causes, such as two areas having a shared structural connectivity (wiring connections) or being driven by a common input. As such, studies based on FC are highly valuable for addressing disruptions of brain functioning in neurological disorders such as epilepsy, a major neurological disorder characterized by chronic unprovoked seizures. Indeed, FC studies in epilepsy are abundant ^2– 7^, but these studies typically perform group comparisons between health and disease; as an alternative to this approach, we present a longitudinal FC analysis on the same mouse brain across different days.

Our general goal here is to unveil whether FC can account for differences in brain states, across the entire transition from a healthy brain to an epileptic one after an initial episode of status epilepticus. To the best of our knowledge, variations in FC across this transition have not addressed before. Our hypothesis is that FC can indeed differentiate those states.

To this aim, we introduce a setup based on a classical animal model of mesial temporal lobe epilepsy (MTLE), achieved by intra-hippocampal injection of kainic acid (KA) ^8, 9^. It is well-known in this model that after an initial provoked seizure, the latent period emerges and eventually, mouse brain’s resembles the main characteristics of human MTLE (see for instance ^10^ and references therein). Using this validated model of epilepsy, we show herein that FC can indeed differentiate those states when applied to longitudinal data.

## Methods

### Experimental protocol

All the experiments were performed employing a C57Bl/6 mouse (The Jackson Laboratory, Sacramento, CA). The animal was housed with ad libitum food and water access, in a 12:12h light cycle. All procedures were approved by the University of the Basque Country EHU/UPV Ethics Committees (Leioa, Spain) and Baylor College of Medicine Institutional Animal Care and Use Committee (Ethical approval number: AN5004; Houston, TX, USA). All animal procedures followed the European directive 2010/63/UE and NIH guidelines.

For this study, an adult mouse (male, 8 weeks old) was subjected to an intra-hippocampal injection of the glutamate agonist kainic acid (KA, 1nmol of KA in 50 nL, Sigma-Aldrich, St Louis, MO, USA), an experimental model that reliably reproduces the physiopathological features observed in human MTLE ^10, 11^.

In brief, the mouse was anesthetized with ketamine/xylazine (10/1 mg/kg) and received a single dose of the analgesic buprenorphine (1mg/kg) subcutaneously. After positioning in the stereotaxic apparatus, a 0.6mm whole was drilled at coordinates taken from Bregma: anteroposterior (AP) -1.7mm, laterolateral (LL) -1.6mm. A pooled glass microcapillary was inserted at -1.9mm dorsoventral (DV), and 50nL of KA (20mM) were delivered into the right hippocampus using a microinjector (Nanoject II, Drummond Scientific, Broomal, PA, USA). After 2min, the microcapillary was retracted, and the mouse sutured and maintained in a thermal blanket until recovered from anesthesia.

### Mouse recordings

The mouse was implanted with intracranial electrodes E363/8 platinum/iridium Teflon insulated (PlasticsOne, Roanoke, VA, USA), 0.005mm in diameter and 2mm in length, mounted in a plastic pedestal, which was secured to the skull with dental cement.

**Figure 1.**
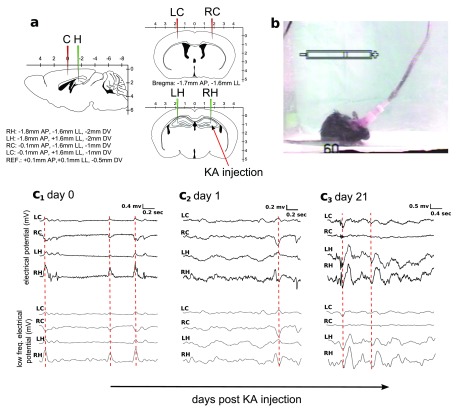
Intracranial EEG recordings from MTLE mice. **a**: Experimental setup. The intracranial placement of site recordings consisted on two electrodes placed bilaterally in the cortex (LC and RC, red) and two in the hippocampus (LH and RH, green). **b**: EEG recording was coupled to videographic recordings for visual confirmation of the seizure events. **c1**- **c3**: Examples extracted from the EEG recordings at the day of the injection ( **c1**), the next day ( **c2**) and after 21 days ( **c3**). Overall changes in the electrical potential are shown in the upper row and after filtering for low frequency (1–14 Hz) in the lower row. The red dotted line marks high statistical similarities between electrodes, what provides high values of FC. Notice that RH is the site of the KA injection, and shows a higher epileptogenic activity that can be easily detected by looking at the amplitude of the time series associated to the RH electrode.

According to Figure 1, four electrodes were implanted bilaterally in the motor cortex and hippocampus. The four electrodes were positioned at -0.1mm AP, +1.6mm LL, -1mm DV (left cortex); -0.1mm AP, -1.6mm LL, -1mm DV (right cortex); -1.8mm AP, +1.6mm LL, -2mm DV (left hippocampus); -1.8mm AP, -1.6mm LL, -2mm DV (right hippocampus). The reference electrode was placed at the frontal lobe at +0.1mm AP, +0.1mm LL, -0.5mm DV, and the ground electrode was positioned over the cervical paraspinous area. Hereon, we labeled these electrodes as left cortex (LC), right cortex (RC), left hippocampus (LH) and right hippocampus (RH). The KA injection was applied at the site of the RH electrode (indicated by a red arrow in panel a).

Recording sessions had a duration of 4 hours and were performed every day during the first week and every other day for the following weeks with a Nicolet video-electroencephalogram (vEEG) system (NicView 5.71, CareFusion, San Diego, CA, USA). Recordings were first preprocessed with a 60 Hz notch filter and then passed through a (0.5–250) Hz bandwidth filter. Next, data was converted to ASCII using an EEG Converter for further analysis (EegSoft, Inc.). All postprocessing analysis was performed in Matlab (MathWorks Inc., Natick, MA).

Changes in FC patterns were analyzed from these intracranial electroencephalographic data (EEG) across longitudinal sessions, from 0 days post KA injection (0 dpi) to 21 dpi.

### Identification of interictal states

Epileptic seizures and artifact-free periods of interictal states were visually classified; seizures were identified according to repetitive-spikes and slow-wave discharges lasting 10 sec or more and synchronized with the behavioral stage 4–5 generalized seizures (monitored by video recording) according to the Racine scale ^12^. Interictal discharges were measured as fast and high amplitude spike events lasting up to 200 msec.

### Functional Connectivity

FC was addressed by calculating the correlation (C) and the partial correlations (PC) matrices between the time series electrode data. To calculate both C and PC, let *x _i_* be a column vector in which rows represent observations (time points) and *i* = *LH,RH, LC,RC* one possible electrode. Here, C and PC were calculated over non-overlapping windows of 1250 time points of interictal activity each (which is equivalent to having time windows of 5 seconds duration, as the sampling frequency was 250 Hz). Then, we build the data set matrix as *X* ≡ [ *x _LC_ x _RC_ x _LH_ x _RH_*], a matrix with dimension 1250 times 4.

**Calculation of C.** Given *X*, each element matrix *C _ij_* is defined as the Pearson’s correlation coefficient between the time series *x _i_* and *x _j_*, with *i, j* = *LH,RH, LC,RC*. Here, C was calculated using the *corr* function in Matlab (MathWorks Inc., Natick, MA). In particular, we run *C* = *corr*( *X*), which returns a matrix with dimension 4 times 4. Each element matrix satisfies that -1 ≤ *C _ij_* ≤ 1, with high and positive *C _ij_* meaning that the two time series *x _i_* and *x _j_* are correlated, high and negative values means anticorrelated and *C _ij_* ≈ 0 that the two time series are statistically uncorrelated, ie., independent.

**Calculation of PC.** Given *X* and assuming C to be an invertible matrix, each element PC _*ij*_ is defined as $-\frac{P_{ij}}{\sqrt{P_{ii}P_{jj}}},$ where *P* ≡ C ^-1^ is the inverse of the correlation matrix (ie. the so-called precision matrix). Notice that again by construction of PC, we have -1 ≤ PC _*ij*_ ≤ 1. Here, PC was calculated using the *partialcorr* function incorporated in Matlab (MathWorks Inc., Natick, MA), running the code *PC* = *partialcorr*( *X*), which similar than C has a dimension of 4 times 4.

It is important to emphasize that when comparing C to PC, high values of C _*ij*_ are possible due to the presence of common neighbors to both *i* and *j*, ie., coming from *z* ≠ *i*, *j*, but PC removes that correlation contribution coming from those other neighbors ^13^.

**FC across days.** For the following days: 0,1,2,7,14 and 21 dpi, we averaged both C and PC over eight different non-overlapping windows of size 1250 time points each. FC values (mean and standard deviation) were calculated for all days. The raw data corresponding to the eight time windows and all the different days are available below ( Dataset 1– Dataset 6).

Raw intracranial data for recording day dpi0 Four electrodes were implanted bilaterally in the motor cortex and hippocampus. Label electrodes were LC (left cortex), RC (right cortex), LH (left hippocampus) and RH (right hippocampus). These data corresponds to days post KA injection (dpi) equal to 0 (ie. the same day of the injection). There are a total number of 8 files (window1, window2,...,window8), each one corresponding to one independent time window (ie., a segment) of 1250 time points of interictal activity. Thus, each of these files have 1250 time points in rows and 4 columns, each one containing the electrical potential in mV for each of the electrodes (LC,RC,LH,RH) ^17^. Click here for additional data file. Copyright: © 2015 Erramuzpe A et al. 2015 Data associated with the article are available under the terms of the Creative Commons Attribution Licence, which permits unrestricted use, distribution, and reproduction in any medium, provided the original data is properly cited.

Raw intracranial data for recording day dpi1 As for file dpi0.xls but for dpi1 ^18^. Click here for additional data file. Copyright: © 2015 Erramuzpe A et al. 2015 Data associated with the article are available under the terms of the Creative Commons Attribution Licence, which permits unrestricted use, distribution, and reproduction in any medium, provided the original data is properly cited.

Raw intracranial data for recording day dpi2 As for file dpi0.xls but for dpi2 ^19^. Click here for additional data file. Copyright: © 2015 Erramuzpe A et al. 2015 Data associated with the article are available under the terms of the Creative Commons Attribution Licence, which permits unrestricted use, distribution, and reproduction in any medium, provided the original data is properly cited.

Raw intracranial data for recording day dpi7 As for file dpi0.xls but for dpi7 ^20^. Click here for additional data file. Copyright: © 2015 Erramuzpe A et al. 2015 Data associated with the article are available under the terms of the Creative Commons Attribution Licence, which permits unrestricted use, distribution, and reproduction in any medium, provided the original data is properly cited.

Raw intracranial data for recording day dpi14 As for file dpi0.xls but for dpi14 ^21^. Click here for additional data file. Copyright: © 2015 Erramuzpe A et al. 2015 Data associated with the article are available under the terms of the Creative Commons Attribution Licence, which permits unrestricted use, distribution, and reproduction in any medium, provided the original data is properly cited.

Raw intracranial data for recording day dpi21 As for file dpi0.xls but for dpi21 ^22^. Click here for additional data file. Copyright: © 2015 Erramuzpe A et al. 2015 Data associated with the article are available under the terms of the Creative Commons Attribution Licence, which permits unrestricted use, distribution, and reproduction in any medium, provided the original data is properly cited.

### Network Connectivity Index

**Figure 2.**
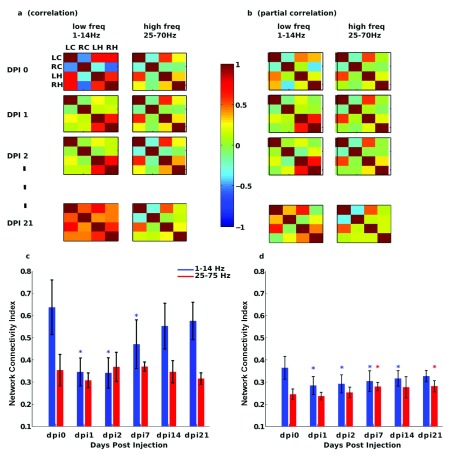
Longitudinal variations of FC across different days and different frequency bands. **a**, **b**: C and PC matrices across different days post KA injection and different frequency bands: low freq (1–14 Hz) and high freq (25–70 Hz). **c**, **d**: For the matrices plotted in panels a and b, we calculated the network connectivity index (for definition see methods) and represented across different days and frequency bands. Asterisks mean, for each condition respect to dpi0 (control), statistical significance differences with pvalue smaller than 0.05. C (and to a smaller extent PC) clearly differentiate brain states across days in the lower frequency band (blue line), showing a strong decrement at dpi1 and afterwards, FC started to increase until dpi21.

Motivated by a previous study of synchronization clusters in human temporal lobe epilepsy ^14^, we introduce here a network synchronization index, we dub the Network Connectivity Index (NCI), that accounts for all electrode interactions, c.f., Figure 2c,d. To calculate NCI, we summed all the absolute values of all matrix elements in either C or PC divided by *N*( *N* - 1), a normalization factor equal to the total number of pairs contained in the sum minus the principal diagonal elements; thus, the NCI ignores all diagonal elements C _*ii*_ and PC _*ii*_, as they are equal to 1 in both C and PC matrices.

For each of the eight non-overlapping windows we calculated one value of NCI. Statistical significance differences between the NCI values at dpi0 (control) in respect to other conditions (1,2,7,14 and 21 dpi) were addressed by performing a paired t-test of the hypothesis that the two data sets (8 values of NCI in each group) have a different mean. Here, the t-test was performed using the *ttest* function incorporated in Matlab (MathWorks Inc., Natick, MA).

### Low and high frequency regimes

Brain electrophysiological signals are well-known to be a mixture of many different rhythms occurring each at a different time scale ^15^; as a consequence, one electrode data contains activity which results from a superposition of different rhythms. Classical Berger’s criteria (see 15 and references therein) separates brain oscillations occurring within different frequency bands (delta 0.5–4 Hz; theta 4–8 Hz; alpha 8–12 Hz; beta 12–30 Hz; gamma > 30 Hz), the higher the frequency, the fastest the rhythm’s oscillations are.

In this study, rather than calculating FC measures directly from the raw data, we first band-pass filtered the data and pooled all the frequency classes in two regimes: one occurring at low frequency bands (1–14 Hz, thus merging together delta/theta/alpha bands) and another one focused on high frequency bands (gamma rhythms at 25–70 Hz). To perform band-pass filtering, we applied a zero-phase digital filter to the input data *X* (ie., the raw data), that depending on the minimum (1 Hz) and maximum (14 Hz) values of frequency to be filtered in, it allows to extract the output data *X _low_*, which contains the 1–14 Hz contribution of the original signal (see panels in Figure 2c _1_–c _3_ for visualization of both *X* and *X _low_*). Similarly, the same bandpass filter applied to *X* but with different minimum (25 Hz) and maximum (70 Hz) frequencies returned *X _high_*. The Matlab code of the used function here (named *BandPassFilter.m*) is available below ( Dataset 7). Notice that *BandPassFilter.m* uses as an input parameter the sampling frequency (here, 250 Hz) and that internally it uses the function *filtfilt*, incorporated in the default Matlab (MathWorks Inc., Natick, MA).

Band Pass Filter A small MATlab function used for data filtering (explained in the manuscript) ^23^. Click here for additional data file. Copyright: © 2015 Erramuzpe A et al. 2015 Data associated with the article are available under the terms of the Creative Commons Attribution Licence, which permits unrestricted use, distribution, and reproduction in any medium, provided the original data is properly cited.

In summary, the FC measures (both C and PC and consequently NCI) were calculated over *X _low_* and *X _high_* rather than on *X*. This selection of frequency bands was performed to show explicit differences in the dynamics between the two highly different regimes.

## Results

The setup represented in Figure 1 provided unique data to test our hypothesis that the FC analysis, when applied to longitudinal data, can differentiate between brain states. Taking as an input the recordings obtained from the four electrodes LC, RC, LH and RH, we calculated FC matrices based on C and PC on eight different time-windows (Methods). In all the days, averaging over the eight windows ensured an appropriate sample with regard to the variability in the FC estimation, as the coefficient variation defined as the ratio between the standard deviation and the mean value was around 0.1 or less.

Figure 2a,b correspond to average C/PC matrices across the eight segments. Based on these values we quantified connectivity patterns by calculating the NCI ( Figure 2c,d) which, by summing the absolute values of all C and PC values (methods), provides information of the overall network connectivity.

The C analysis ( Figure 2a) showed a strong non-linear behavior at low frequencies (left column of matrices), as FC values strongly decreased at 1 dpi and after this point the FC values started to increase up to 21 dpi. This tendency, also observed in the NCI index (blue bars in Figure 2c), did not exist in the high-frequency regime (right column of matrices in Figure 2a and red bars in Figure 2c), confirming that FC significantly varied across brain states, as data come from a well-validated model of mouse epilepsy. Figure 2a also shows that hippocampal electrodes LH and RH remained strongly correlated across the time period in comparison to electrodes in the cortex. In particular, LC and RC started highly anticorrelated at 0 dpi, but their mutual correlation was drastically decreased at 1 dpi to eventually start to increase again up to a highly correlated state at 21 dpi.

The PC analysis in the low frequency regime was also able to differentiate between hippocampal electrodes LH and RH, as the FC value between these two electrodes was high across the experimental period ( Figure 2b). With regard to NCI ( Figure 2d), the PC analysis showed small variations across days in comparison to the C analysis, and this occurred for both high and low frequency regimes ( Figure 2c vs Figure 2d). This has a particular interest, as PC removes interactions in a given pair coming from common neighbors, the so-called indirect effects. Thus, the indirect effects captured by C but not by PC were dominant at low frequencies.

Finally, we analyzed the size effects by computing the Cohen’s d parameter ^16^. Size effects where high in both C/PC NCI indexes. Thus, when comparing the NIC value at dpi0 with other days, respectively, (dpi1, dpi2, dpi7, dpi14 and dpi21), the Cohen’s d parameter based on C results was (2.9797, 2.9716, 1.4273, 0.7480, 0.5870). Similarly, with regard to NCI based on PC, the Cohen’s parameter was (3.3111, 3.2127, 2.9904, 2.9513, 2.8848), which indicated high size effects for both C/PC NCI indexes (the higher the Cohen’s distance, the bigger the size effects).

## Discussion

Can FC differentiate between brain states when applied to longitudinal data? To answer this question, we have made use of an animal model of MTLE to address the variations in FC across the transition from an initial episode of status epilepticus to seizure chronification. We addressed FC by calculating C and PC. C (but not PC) revealed interactions through common neighbors ( *i.e.*, *network effects*) at low-frequency bands. More precisely, the network index for C showed a strong drop-off in the overall brain connectivity at 1 dpi but it smoothly increased for several days afterwards. This tendency might be correlated with the latency period, namely, the time interval between the original brain insult and the clinical presentation of the first spontaneous seizure. During this latency period, the transition from a healthy brain into an epileptic one, or epileptogenesis, occurs due to changes in the molecular, cellular, and network properties of the brain in response to the initial precipitating event. What we particularly show here is that the NCI for C (and to a lesser position PC) works as a readout of the changes in brain functioning that take place during the latency period. In the near future, we aim to correlate the FC results with studies at the molecular and cellular level, what might an integrative approach to better understand the process of epileptogenesis to eventually open new venues for more efficient therapeutic strategies.

We are emphasizing the advantages of using animal models for studying epilepsy. Thus, rather than performing group comparison (health vs. epilepsy) as it is normally done when studying disease, our setup allowed us for addressing longitudinal variations in FC on the same animal from the initial episode of status epilepticus to chronic epilepsy. Although the results analyzed here correspond to a very limited sample (n=1), we firmly believe that the same analysis can be applied to larger samples, allowing for studying longitudinal group FC patterns rather than individual results.

It is tempting to speculate that the increasing synchronization of the network observed after the initial status epilepticus is driven by the reorganization of the circuitry which occurs during the latent period. For instance, synchronization is higher between right and left hippocampus, as is expected from their direct connection via the hippocampal commisure, while it is lower between hippocampus and the primary somatosensorial cortex, as their connection is more indirect through a relay in the entorhinal cortex. Furthermore, our results insinuate that this synchronization is exclusive of low frequency bands (1–14Hz, delta/theta/alpha bands combined together), suggesting an underlying specificity of the hippocampal circuitry. Nonetheless, we are cautious to reach biologically relevant conclusions with n=1 and we will test these hypotheses in the future in larger samples of data. Rather, our data serves as a proof of principle that longitudinal variations of brain functional connectivity detect changes in brain connectivity in the epileptic mouse brain during the reorganization of the hippocampal circuitry after the initial status epilepticus.

In summary, FC calculated from intracranial electroencephalography might work as a readout of brain functioning and provides a straightforward measure for studying the effect of biological alterations occurring at the molecular, cellular and physiological scales during the transition from a healthy brain into an epileptic one. Importantly, the FC analysis presented here is based on longitudinal recordings of the same experimental subject allowing a continuous and precise temporal resolution that makes the calculation of FC meaningful and robust. Finally, it is important to remark that our analysis, bringing together experimental, mouse-based disease, biological science and computational science, might help to pave the road for further, more elaborated, computational algorithms as tools for analysis and validation of other diseases.

## Data availability

The data referenced by this article are under copyright with the following copyright statement: Copyright: © 2015 Erramuzpe A et al.

Data associated with the article are available under the terms of the Creative Commons Attribution Licence, which permits unrestricted use, distribution, and reproduction in any medium, provided the original data is properly cited. 

*F1000Research*: Dataset 1. Raw intracranial data for recording day dpi0, 10.5256/f1000research.6570.d48989

*F1000Research*: Dataset 2. Raw intracranial data for recording day dpi1, 10.5256/f1000research.6570.d48990

*F1000Research*: Dataset 3. Raw intracranial data for recording day dpi2, 10.5256/f1000research.6570.d48991

*F1000Research*: Dataset 4. Raw intracranial data for recording day dpi7, 10.5256/f1000research.6570.d48992

*F1000Research*: Dataset 5. Raw intracranial data for recording day dpi14, 10.5256/f1000research.6570.d48993

*F1000Research*: Dataset 6. Raw intracranial data for recording day dpi21, 10.5256/f1000research.6570.d48994

*F1000Research*: Dataset 7. Band Pass Filter, 10.5256/f1000research.6570.d48995
