# Supplementary material for: Longitudinal variations of brain functional connectivity: A case report study based on a mouse model of epilepsy
Source: F1000Res. 2015 Jul 16;4:144. Originally published 2015 Jun 5. [Version 2] doi: 10.12688/f1000research.6570.2 (PMC4482210; doi:10.12688/f1000research.6570.2)
Supplement: Peer review history

# Peer review: Referee response for version 2

**DOI:** 10.5256/f1000research.7306.r9528

**Published:** 2015-07-16

**Authors:** Roma Siugzdaite

**Affiliations:** 1 Department of Data Analysis, Ghent University, Ghent, Belgium

They have read the answers to my comments, the appropriate corrections to the comments were made.

I have read this submission. I believe that I have an appropriate level of expertise to confirm that it is of an acceptable scientific standard.

# Peer review: Referee response for version 1

**DOI:** 10.5256/f1000research.7055.r9189

**Published:** 2015-06-24

**Authors:** Ruedi Stoop

**Affiliations:** 1 Institute of Neuroinformatics, ETH Zürich, Zürich, Switzerland

This manuscript provides evidence that for studying brain diseases, the use of animal models for brain connectivity analysis is opening promising avenues. In an animal model case study, the authors analyze here in the same brain the transition from health to disease. Their methodology reveals an important link between molecular and large-scale brain imaging. Despite the convincing results the authors obtain, the reader should be aware that these results, although methodologically consistent, are in need of further validation, as they are based on an isolated one-animal study.

I have read this submission. I believe that I have an appropriate level of expertise to confirm that it is of an acceptable scientific standard.

# Peer review: Referee response for version 1

**DOI:** 10.5256/f1000research.7055.r8921

**Published:** 2015-06-15

**Authors:** Roma Siugzdaite

**Affiliations:** 1 Department of Data Analysis, Ghent University, Ghent, Belgium

The authors investigated functional connectivity (FC) changes during different time points in one mouse brain with the lesion to hippocampus. The study is very interesting and informative in several ways:

1. they tracked longitudinal variations of FC through 3 weeks after the lesion was induced;
2. they investigated three different stages of epilepsy;
3. they introduced a network synchronization measure NCI which takes into account interactions between all electrodes;
4. they divided frequencies into low (1-14Hz) and high (25-70Hz) frequency bands, in which they found interactions revealed through common neighbours;
5. NCI was reflecting Correlation results in low frequency band.

Even though the study is the first step to understand dynamics and (probably in the future the mechanism) of epilepsy using functional connectivity tools, I have few concerns.

1. First of all since it is a case study, we are looking only at one animal data. We have to keep it in mind.
2. The three time points: initial insult, latent period and chronic epilepsy are described as the interval of interest. Identification of interictal states are described in methods, but in the results this information was not taken into account. How number of seizures were distributed in different stages and how it could influence changes in FC?
3. In the experimental protocol part when the injection is explained there is a mistake "saline or KA were delivered". It was only one mouse, so I suppose that only Kainic Acid was injected (and not saline, was mostly used for control condition).
4. Since T-test is performed on a small sample size, it would be good to calculate also the effect size.
5. In the results part the sentence starts "We analysed NCI across brain regions..." and then authors talk about correlations from electrodes in hippocampus, that is in C analysis, but not in NCI.
6. Personally for me the results are very interesting, especially at DPI 0. We would expect to see differences in connections RH to RC, and from there RH with other regions in case of hippocampus damage, but it's not described in the discussion at all.
7. Methodologically paper is very good, though some physiological explanations or hypothesis in discussion would be useful. For example, why correlations remain strong despite the lesion in RH with LH  in low freq; and between LH and LC in high frequency?

This article is acceptable, although these minor corrections should be made.

I have read this submission. I believe that I have an appropriate level of expertise to confirm that it is of an acceptable scientific standard.

# Response

**Published:** 2015-07-07

**Authors:** Jesus Cortes

**Affiliations:** Biocruces Health Research Institute, Spain

1. *First of all since it is a case study, we are looking only at one animal data. We have to keep it in mind.*
  We fully agree with the reviewer that these results are based on a single animal study. Indeed, this fact has been stated explicitly in several occasions throughout the text. Therefore, we are aware that these results need for a further validation. However, we really would like to emphasize that what we are presenting here is a new methodologically setup to tackle brain connectivity on same brain in the transition from a healthy to pathological brain.
2. *The three time points: initial insult, latent period and chronic epilepsy are described as the interval of interest. Identification of interictal states are described in methods, but in the results this information was not taken into account. How number of seizures were distributed in different stages and how it could influence changes in FC?*
  We agree with the reviewer that it would be very interesting to correlate the number of seizures with the FC across the different stages (initial insult, latent period, chronic epilepsy); however, our methodological approach does not allow us to properly estimate the number of seizures per day when seizures are less frequent because of the low sampling (4h recording session per day). To avoid misguiding the readers, we have put less emphasis in these three periods in the abstract, and incorporated the new sentence:
  “To this end, we present a case report study based on a mouse model for epilepsy and analyze longitudinal intracranial electroencephalography data of epilepsy to calculate FC changes from the initial insult (status epilepticus) and over the latent period, when epileptogenic networks emerge, and at chronic epilepsy, when unprovoked seizures occur as spontaneous events.”
3. *In the experimental protocol part when the injection is explained there is a mistake "saline or KA were delivered". It was only one mouse, so I suppose that only Kainic Acid was injected (and not saline, was mostly used for control condition).*
  Thanks to the reviewer for this comment. We have corrected this error and removed “saline”.
4. *Since T-test is performed on a small sample size, it would be good to calculate also the effect size.*
  We want to thank the reviewer for making us to clarify this. We have calculated the size effects by computing the Cohen’s d parameter ( Cohen, 1988).  When comparing the NIC value at dpi0 with other days, respectively, (dpi1, dpi2,dpi7, dpi14 and dpi 21), the Cohen’s d parameter based on C results was (2.9797,    2.9716,    1.4273,    0.7480,    0.5870). Similarly, for NCI based on PC, we found (3.3111,    3.2127,    2.9904 ,   2.9513 ,   2.8848), which indicated high size effects for both C/PC NCI indexes (the higher the Cohen’s distance, the bigger the size effects and in any case the size effects were high).
  This text has been added in the final paragraph of Results in the revised version.
  We also added a new reference:
  Cohen, J. (1988). Statistical power analysis for the behavioral sciences (2nd ed.). Hillsdale, NJ: Lawrence Earlbaum Associates.
5. *In the results part the sentence starts "We analysed NCI across brain regions..." and then authors talk about correlations from electrodes in hippocampus, that is in C analysis, but not in NCI.*
  We thank the reviewer for this comment. We now edited the text and corrected this.
6. *Personally for me the results are very interesting, especially at DPI 0. We would expect to see differences in connections RH to RC, and from there RH with other regions in case of hippocampus damage, but it's not described in the discussion at all.*
  We thank the reviewer for this insight. Sample size, n=1, makes difficult to generalize; however, we added the following paragraph in Discussion, as suggested by the reviewer:
  “It is tempting to speculate that the increasing synchronization of the network observed after the initial status epilepticus is driven by the reorganization of the circuitry which occurs during the latent period. For instance, synchronization is higher between right and left hippocampus, as is expected from their direct connection via the hippocampal commisure, while it is lower between hippocampus and the primary somatosensorial cortex, as their connection is more indirect through a relay in the entorhinal cortex. Furthermore, our results insinuate that this synchronization is exclusive of low frequency bands (1-14Hz, delta/theta/alpha bands combined together), suggesting an underlying specificity of the hippocampal circuitry. Nonetheless, we are cautious to reach biologically relevant conclusions with n=1 and we will test these hypotheses in the future in larger samples of data. Rather, our data serves as a proof of principle that longitudinal variations of brain functional connectivity detect changes in brain connectivity in the epileptic mouse brain during the reorganization of the hippocampal circuitry after the initial status epilepticus.”
7. *Methodologically paper is very good, though some physiological explanations or hypothesis in discussion would be useful. For example, why correlations remain strong despite the lesion in RH with LH  in low freq; and between LH and LC in high frequency?*
  This is indeed an interesting observation but difficult to be extrapolated any interpretation based on only n=1 data set. It is well known, as it is acknowledged now in the manuscript, that different electrophysiological rhythms have different behavioral and cognitive correlates, and the fact that hippocampus electrodes are much higher correlated at slow rhythms and cortex electrodes do correlate at high rhythms, although very striking, needs more data to have a clearer interpretation.
  This point also is related to previous concern, number 6.
